# HBV Perinatal Transmission

**DOI:** 10.1155/2013/875791

**Published:** 2013-04-22

**Authors:** Muhammad Umar, Hamama-tul-Bushra, Shifa Umar, Haider Ali Khan

**Affiliations:** ^1^Rawalpindi Medical College, Holy Family Hospital, 965-B, Saidpur Road, Satellite Town, Rawalpindi 46000, Pakistan; ^2^Rawalpindi Medical College, Rawalpindi 46000, Pakistan; ^3^Shifa International Hospital, Islamabad 44000, Pakistan; ^4^Department of Medicine, Centre for Liver and Digestive Diseases, Holy Family Hospital, Rawalpindi 46000, Pakistan

## Abstract

Hepatitis B is a serious public health problem all around the world. It is a blood-borne and sexually transmitted DNA virus in adults, but mother to child transmission of hepatitis B virus also occurs in infants born to hepatitis B surface antigen positive mothers.

## 1. Epidemiology

About 350 million individuals have chronic HBV infection worldwide, and half of them acquired the infection either through perinatal route or in early childhood [1, 2], the latter being mostly asymptomatic, frequently goes unrecognized, yet 30–90% of them will develop chronic infection, which will not be diagnosed until adulthood [3–7].

## 2. Modes of HBV Transmission in Infants and Children

The modes of HBV transmission at an early age are (i) perinatal transmission, (ii) vertical transmission from an infected mother to her infant, or (iii) horizontal transmission from an infected household contact to the child [8].

## 3. Risk Factors for Mother to Child Transmission (MTCT)

The presence of HBeAg in serum is an indicator of viral replication. It is often used as a marker of ability to spread the virus to other people. HBeAg can filter through the placenta and can infect the fetus. Mother to child transmission rate is about 70–90% for HBeAg-positive mothers, 25% for HBeAg-negative/HBeAb-negative mothers, and 12% for HBeAg-negative/anti-HBe-positive mothers [9–13]. While there is only 6.6% transient infection in infants born to HBeAg-positive mothers, no persistent infection has been documented. 

High level of HBV DNA is the main risk factor for perinatal transmission in pregnant women. HBV can infect the placental tissue and vascular endothelium. HBV DNA is also present in sperms of HBV-infected males, and homology between the father and child's viral sequences has been found [12, 14].

HBV can infect the follicular fluid and ovary, and studies have suggested that high levels of maternal HBV DNA enhance HBV transmission to embryos. Nie et al. demonstrated HBV replication in 50% embryos from mothers with HBV viremia versus 7% in mothers with undetectable levels of HBV DNA [15–17].

As regards the HBV genotype, some Japanese studies showed association between genotype C and MTCT when compared with other genotypes which were refuted by some later Chinese studies. Similarly the role of precore mutant (HBeAg-negative) and basal core mutation is also not very well documented in MTCT. Therefore, no conclusive evidence can be drawn regarding association of HBV genotypes (A, B, C, and D) and perinatal transmission [16–19].

## 4. Routes for Mother to Child Transmission (MTCT)

*Intrauterine transmission* is the detection of HBsAg or HBV DNA in neonatal peripheral venous blood or cord blood. It may occur either from sperms or maternal oocytes in early embryonic stage. Mother to child transmission during intrauterine life occurs through maternal blood cells to cells in the placenta or by placental leakage at the time of preterm labor [17, 20].

*Intrapartum transmission* has strong association with duration of the first stage of labour lasting >9 hours. Partial placental leakage and trauma due to instrumentation during labour increase the rate of MTCT due to mixing of fetal and maternal blood (microtransfusion) [21].

There is no convincing evidence that *postpartum transmission* occurs due to ingested vaginal secretion at the time of birth, although the HBsAg was detectable in 90% of gastric lavage fluid of infants born to infected mothers, probably due to intact oral and gastric mucosa [22]. 

Post partum HBV MTCT with breastfeeding is also controversial. HBsAg was detected in 72% of breast milk samples and can be transmitted especially if mothers had abrasion on nipple. However, the published data does not support the risk of transmission through this route [23].

## 5. Screening for HBV Infection during Pregnancy

There is no consistent universal policy for screening HBV infection in pregnancy. Also, universal testing has financial implications in developing countries of Asia and Africa and is suggested in high-risk mothers only. HBsAg testing should be done at the first prenatal visit with other recommended screening tests. US Preventive Service Task Force (USPSTF) has recommended HBsAg testing in every pregnant woman regardless of previous testing or vaccination at the time of hospital admission or other delivery setting; women with unknown HBsAg status or with new or continuing risk factors for HBV infection should be screened again at the time of delivery [24].

In Europe, many countries rely on risk factors to determine indication of screening as there is no uniform policy for HBV testing during pregnancy. However, a recent report from Denmark indicated that approximately 50% of infected pregnant women would not have been identified using this strategy [25, 26]. The changing immigration patterns in Europe and USA also favor a more general or universal screening and immunization program [27]. 

Screening is the main key factor in the successful prevention and control of HBV infection. Thus, keeping in view the high perinatal HBV transmission rate, it is recommended that all pregnant women should be tested for HBsAg at the time of first antenatal visit and followed accordingly. Screening for HBV infection during pregnancy is the most effective way to identify newborns that require prophylaxis with hepatitis B vaccine and HBIG as well as pregnant women who require antiviral therapy. 

## 6. Prevention of Mother to Child Transmission (MTCT)

### 6.1. Prevention *In Utero* and during Delivery

Although neonatal immunization may result in 75% to 90% reduction of the HBV carrier rate [28–31], at least 10% of the cases of HBV transmission cannot be prevented by this method. According to literature, 88% of the children with breakthrough infection had HBsAg positive mothers with HBeAg-positive status [1, 12, 30–36]. The explanation for this high risk of transmission for HBeAg-positive mother is the inability of HBeAg to enter fetal circulation via placenta and to induce T-cell tolerance *in utero* [37]. The maternal HBeAg-positive status and a high HBV DNA load are strongly associated with intrauterine transmission [38]. Decreasing maternal HBV DNA viral load might thus be an effective way to reduce the rate of HBV infection in infants.

### 6.2. Role of Antiviral Therapy in Prevention of MTCT

Pregnant women with chronic hepatitis B should have baseline evaluations including hepatitis B surface antigen (HBsAg), HBeAg, antibody to HBeAg (anti-HBe), HBV DNA, and serologic markers for the presence of other viral infections [39]. Mothers with serious abnormalities in liver functions are more prone to maternal (postpartum hemorrhage, puerperal infection) as well as fetal (low body weight infants, fetal distress, premature birth, fetal death, and neonatal asphyxia) complications. Regular monitoring of liver function and HBV DNA level should be performed in the gestational period to determine (i) the progression of liver disease and (ii) the need for antiviral therapy [40]. 

The main complications associated with cirrhotic liver disease in pregnancy are rupture and bleeding from esophageal varices (20%–25%). They mostly occur especially during the second trimester or labor. Endoscopic band ligation is the standard treatment option for women planning a pregnancy in the presence of known esophageal varices [41]. Other options like shunt surgery or even liver transplantation before pregnancy should also be considered. All patients should undergo upper endoscopy for assessment of varices in the second trimester. Beta blocker therapy is mandatory for large varices, despite concerns of occasional fetal effects [42]. 

A number of factors influence treatment option in women of childbearing age. The most important aspect to consider is the safety of antiviral drugs in pregnancy and breastfeeding. Other factors include agent's efficacy and its barrier to resistance and proposed length of therapy. Therapy can be delayed if pregnancy is planned in near future. A careful assessment of the degree of hepatic activity and fibrosis, with either liver biopsy or noninvasive method, is required in these cases [43, 44]. In cases of pregnant patients with established liver cirrhosis, therapy with oral antiviral agents (lamivudine, tenofovir, or telbivudine) is advised along with regular monitoring [45]. 

Interferon is contraindicated during pregnancy, and nonpregnant women taking Interferon-based antiviral regimen are advised to use strict contraception during treatment. It may also be stressed that pregnancy should be planned at least 6 months after discontinuation of interferon. Interferon can be used due to a defined period (48–96 wk) of treatment, and a high chance of clinical remission is expected along with HBeAg seroconversion [46]. 

Lamivudine treatment is also safe and effective for chronic HBV-infected pregnant women in early pregnancy or perinatal period, and there are no complications or adverse events associated with it. It has also got no effect on fertilization or embryonic development, and there is no evidence of increase in the incidence of congenital abnormalities in infants. Importantly, it enhances the blocking rate of mother to infant transmission [47].

The therapeutic effect of lamivudine in late pregnancy for the interruption of mother to child transmission (MTCT) of hepatitis B virus (HBV) is now well documented. In a recent meta-analysis conducted by Han et al., compared with controls (placebo or no treatment), lamivudine treatment for the HBV-carrier mothers significantly interrupted MTCT. The efficacy (RR, 95% CI) of lamivudine treatment versus control in 8 RCTs was 0.43, (0.25–0.76); *P* < 0.01, with significant heterogeneity (*P* = 0.04, *I* ^2^ = 52%) as indicated by serum HBsAg. If HBsAg is used as a sole indicator, lamivudine treatment could not significantly interrupt MTCT in the mothers with a viral load >10^8^ copies/mL (before treatment) or in those with a viral load >10^6^ copies/mL (after the treatment). However, lamivudine significantly interrupted MTCT if newborn HBV DNA was used as an indicator with (viral loads criteria as before) [48]. 

In a recent article by Jiang et al., the therapeutic efficacy and safety of lamivudine treatment in late pregnancy were evaluated by analyzing the maternal-fetal outcomes of chronic hepatitis B (CHB) mothers featuring hepatitis B e antigen-(HBeAg-) positivity and highly viremic status. The lamivudine-treated group in contrast to control group had better virological response (97.56%), higher ALT normalization rate (90.20% versus controls: 55.88%; *χ* ^2^ = 13.349, *P* < 0.001), and significantly lower HBeAg titer (957.73 ± 458.42 versus controls: 1296.35 ± 383.14 S/CO; *t* = −5.410, *P* < 0.001). At birth, the infants from lamivudine-treated mothers had significantly lower HBsAg-positivity (15.24% versus controls: 30.43%; *χ* ^2^ = 8.284, *P* = 0.004). Importantly, none of the infants born to lamivudine-treated mothers tested positive for HBsAg by 7–12 months after birth, compared to 8.70% of the infants born to mothers in the control group (*χ* ^2^ = 14.721, *P* < 0.001) [49].

Although lamivudine is a pregnancy category C drug, it still has a relatively high safety profile. Lamivudine treatment for HBV carrier mothers should be initiated at week 28 of gestation. For the HBV carrier mothers with viral load >10^8^ copies/mL, antiviral treatment with lamivudine alone might be not enough to interrupt MTCT. MTCT might be efficiently interrupted if maternal viral load is decreased to the level of <10^6^ copies/mL by lamivudine treatment.

Lamivudine is classified as FDA pregnancy risk category B. In 2011, State Food and Drug Administration approved its use as antiviral drug for CHB. In comparison to lamivudine, it has demonstrated faster and better efficacy in patients with HBeAg-positive and HBeAg-negative CHB disease [50]. In a recent meta-analysis by Deng et al., the pooled results clearly showed significantly lower seropositivity rate for HBsAg or HBV DNA in the telbivudine group (both at birth and at 6–12 months of followup). Maternal HBV DNA levels before delivery were also significantly lower in the telbivudine group [51]. It has also been shown to be safe and effective for entire length of pregnancy with 100% success rate for blocking MTCT [52]. 

In a prospective and open-labeled study evaluating safety of telbivudine, a marked reduction in serum HBV DNA and hepatitis B e antigen (HBeAg) levels along with normalization of elevated ALT levels before delivery was observed with telbivudine treatment. HBV DNA levels start to decline by week 4 and were sustained at a low level after week 12. 33% telbivudine-treated mothers and none (0%) of the untreated controls had PCR-undetectable viremia (DNA < 500 copies/mL) at delivery. The incidence of perinatal transmission was lower in the infants that completed followup born to the telbivudine-treated mothers after seven months of delivery (0% versus controls: 8%; *P* = 0.002). The above-mentioned data supports the use of telbivudine in this special population [36]. In conclusion, telbivudine used during pregnancy is well tolerated and can safely reduce perinatal HBV transmission in CHB HBeAg+ and highly viremic mothers, with no safety concerns in the telbivudine-treated mothers or their infants on short-term followup.

Tenofovir is another antiviral drug (category B in pregnancy) with high efficacy and resistant barrier against HBV infection. It is not yet widely studied but definitely will be more effective in reducing HBV MTCT along with a good safety profile [45].

Keeping in view the above data, quantification of HBV DNA is recommended in all infected women at the end of second trimester at 26–28 week of gestation. If the viral load is >10^6^ copies/mL, antiviral prophylaxis can be started early in the third trimester. In the absence of active liver disease or cirrhosis at base line, treatment can be discontinued 4 weeks after delivery. Breast feeding is not recommended during antiviral treatment [45, 46].

### 6.3. Mode of Delivery

There is no consensus on the issue of whether different mode of delivery will affect the risk of mother to child HBV transmission. Yang et al. [53] performed a meta-analysis of four randomized trials involving 789 pregnant women. The results favored elective caesarian section (ECS) over vaginal delivery due to effective reduction in the rate of mother to child transmission of HBV in the former (ECS 10.5% versus vaginal delivery 28.0%), however, the conclusion of this review must be considered with great caution due to high risk of bias in each study. Furthermore, there were limitations in these studies as per description of methodology used and category C classification of trials. Therefore, ECS is not recommended to prevent MTCT except in individual cases with high HBV DNA load or coexisting HIV infection.

### 6.4. Management of Neonates

Administration of immunoprophylaxis to newborn clearly reduces the rate of perinatal HBV transmission. In a meta-analysis by Lee et al., the relative risk of neonatal HBV infection after HBV vaccine administration was 0.28 (95% (CI) 0.2–0.4) compared with those who received no vaccination. The addition of HBIG to the vaccine further reduced the relative risk 0.54 (95% (CI) 0.41–0.73) when compared to vaccination alone [54].

**Figure 1:**
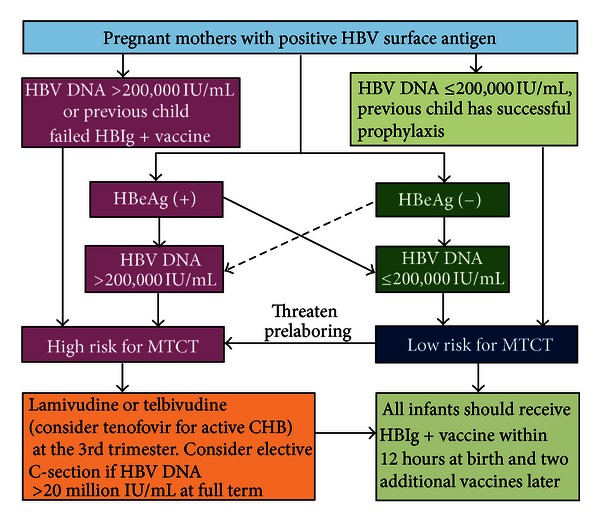
Algorithm for risk assessment and prevention of MTCT of HBV. Adopted from Clinical Gastroenterology and Hepatology 2012.

For new born infants of HBsAg-positive/HBeAg-positive mothers (high risk infants), HBV vaccination and HBIG should be administered within 24 hours after birth followed by two additional vaccine doses (at 1 and 6 months) (Figure 1). The timing of the first dose of vaccine in relation to birth is the most important factor in determining the efficacy of vaccination while increasing the interval between the first 2 doses has little effect on final antibody concentration [55, 56]. Efficacy is optimal when the vaccine is administered within 12–24 hours after birth but declines over next 24 hours and thereafter. The universally agreed recommendation for birth dose is “as soon as possible after birth” (preferably within 24 hours) [57–61].

For new born infants of HBsAg-positive and HBeAg-negative mothers (low-risk infants), the risk of chronic infection is <10%. According to the recommendation of Advisory committee on Immunization Practice (ACIP), USA, the infants born to HBsAg-positive mothers should receive single-antigen hepatitis B vaccine and HBIG within 12 hours of birth administered at different injection sites. The vaccine series should be completed according to the recommended schedule. The final dose of vaccine should not be administered before 24 weeks, age [58]. 

Women with unknown HBsAg status at the time of delivery should have blood drawn and tested as soon as possible after admission. While test results are pending, all infants born to these women should receive the first dose of single-antigen hepatitis B vaccine (without HBIG) within 12 hours of birth. If the mother turns out to be HBsAg-positive, the child should receive HBIG as soon as possible not later than seven days of birth. The rest of the schedule dose should be completed accordingly. Infants born to HBsAg-negative mothers should receive single-antigen hepatitis B vaccine intramuscularly before discharge from the hospital and should complete the series accordingl to the schedule [61]. 

### 6.5. Timely Administration of Birth Dose Vaccine

Administration of HBV vaccine within 24 hours of birth looks promising in a hospital or at a health care setting but in remote areas of developing countries with high HBV prevalence and poor health care infrastructure, timely administration of birth dose is almost impossible. According to a Chinese nationwide survey conducted in 1999, only 38.9% children had received the birth dose within 24 hours of birth. Among children born at home, timely administration of first dose was even lower (maximum 16.7%). In certain regions of China, Indonesia, and Vietnam, different strategies were adopted to improve the timely administration of birth dose, including out of the cold chain (OCC) use of vaccine use of a prefilled monodose, auto-disable device (uniject). Efforts have also been made to increase the awareness of these strategies [8, 62, 63].

The usual route of HBV vaccine administration is intramuscular but it was suggested that intradermal vaccine injection might improve the immunogenicity due to enhanced T-cell response as well as prolonged persistent of HBsAg in skin. An accelerated vaccination schedule also improves the immunogenic response but is not supported by meta-analysis [64].

### 6.6. Low Birth Weight and Preterm Infants

A lower antibody response to HBV vaccination in preterm infants (birth weight < 2000 g) born to HBsAg-negative mothers initially leads the American Academy of Pediatrics (AAP) to recommend deference of HBV vaccine first dose until they reached 2000 g or 2 months of age [53–55]. Subsequent studies in the United States, Europe, and Middle East indicated that three doses of HBV-vaccine provide protective levels of HBsAb, that are comparable to full term infant. The current AAP and ACIP recommendations for preterm newborns includes screening for HBsAg status of the mother and administering the birth dose plus HBIG to all new born who are at risk of HBV exposure [60, 65]. If mother is HBsAg-positive or if no maternal screening is available, the first HBV vaccine dose should be administered at birth and a total of 4 HBV vaccine doses are recommended, only in low risk infants with birth weight <2000 g, the birth dose may be deferred to 30 days of age. WHO recommends that even if the birth weight is <2000 g, the birth dose should still be given within 24 hours but should not be counted towards the primary series and three additional doses should be given [61].

### 6.7. Breast Feeding and HBV MTCT

Beasley et al. compared rate of HBV transmission among breastfed and nonbreastfed babies of HBsAg-positive mothers (53% versus 60%) which markedly reduced with administration of immuneoprophylaxis (0% versus 3%) [23]. It is also documented that in addition to HBsAg, HBeAg and HBV DNA are also present in breast milk. In addition, both clostral HBsAg and HBeAg titers correlate with levels in maternal blood [66–68]. In another study, HBV DNA was found in 81.25% of Chinese mothers (HBsAg and HBeAg positive) and in 45.24% (HBeAg-negative) [69]. Several studies from USA, Taiwan, China, and Italy have reported that breast feeding of HBV-infected mothers carries no additional risk of mother to child transmission [70–72]. Similarly no correlation is found between MTCT and duration of breast feeding. In infants receiving HBIG and HBV vaccine, WHO and American Academy of Pediatrics have also recommended that breast feeding is not a contraindication [73]. In authors opinion, before embarking on any formal recommendations, further reviews and meta-analysis are required on this issue.
